# ^1^H, ^13^C, ^15^N and ^31^P chemical shift assignment of the first stem-loop Guanidine-II riboswitch from *Escherichia coli*

**DOI:** 10.1007/s12104-025-10217-6

**Published:** 2025-02-01

**Authors:** Tatjana Koob, Silas Döpp, Harald Schwalbe

**Affiliations:** 1 Institute for Organic Chemistry and Chemical Biology, Johann Wolfgang Goethe-University Frankfurt, Max‑von‑Laue‑Str. 7, 60438 Frankfurt/M, Germany; 2 Center for Biomolecular Magnetic Resonance (BMRZ), Johann Wolfgang Goethe-University Frankfurt, Max‑von‑Laue‑Str. 9, 60438 Frankfurt/M, Germany

**Keywords:** Regulatory RNA, Riboswitch, Guanidine-II, Solution NMR spectroscopy

## Abstract

A comprehensive understanding of RNA-based gene regulation is a fundamental aspect for the development of innovative therapeutic options in medicine and for a more targeted response to environmental problems. Within the different mechanisms of RNA-based gene regulation, riboswitches are particularly interesting as they change their structure in response to the interaction with a low molecular weight ligand, often a well-known metabolite. Four distinct classes of riboswitches recognize the very small guanidinium cation. We are focused on the Guanidine-II riboswitch with the *mini-ykkC* motif. We report here the assignment of the ^1^H, ^13^C, ^15^N and ^31^P chemical shifts of the 23 nucleotide-long sequence of the first stem-loop of the Guanidine-II riboswitch aptamer from *Escherichia coli*. Despite its small size, the assignment of the NMR signals of this RNA proved to be challenging as it has symmetrical base pairs and palindromic character.

**Supplementary Information:**

The online version contains supplementary material available at 10.1007/s12104-025-10217-6.

## Biological context

Regulatory RNA elements play an important role in the control of gene expression (Nahvi et al. 2002; Parmar et al. 2024). In the non-translated regions of bacterial *m*RNA, one of these RNA elements, riboswitches, change their structure depending on the presence or absence of low molecular weight ligands (Winkler and Breaker 2003). Riboswitches can control the expression of genes at the level of transcription, translation or *m* RNA stability. The widespread abundance of these riboswitches indicates a central and archaic function of guanidine in the metabolism (Breaker et al. 2017). One of the ligands is the cation guanidine. Four different guanidine-dependent riboswitch classes have been identified (Nelson et al. 2017; Sherlock et al. 2017; Sherlock and Breaker 2017; Salvail et al. 2020; Lenkeit et al. 2020). Bacteria can utilize guanidine as a source of nitrogen (Sinn et al. 2021) while at the same time providing protection against possible guanidine toxicity in the cells (Higgins et al. 2019).

In a previous study (Schamber et al. 2022), we have investigated the structural and dynamic properties of the Guanidine-II (Gdn-II) riboswitch, specifically from *Escherichia coli* (*E. coli*). The Gdn-II riboswitch (Sherlock et al. 2017; Huang et al. 2017; Reiss and Strobel 2017) is characterized by the mini-ykkC motif which has two interconnected GC-rich stem-loop structures, P1 and P2. Both helices are closed by the ACGR loop motif which plays a crucial role in the stabilization of the structures and the function of the riboswitch. The riboswitch contains different binding sites for the cooperative binding of either two guanidinium ions (Gdm^+^) (Sherlock et al. 2017) or comparable bivalent ligands (Huang et al. 2019; Steuer et al. 2024). The kissing loop interaction constitutes the fundamental basis of this translational riboswitch (Reiss and Strobel 2017).

To enable further NMR spectroscopic studies of stem-loop P1 capped by a ACGA loop, we report here a nearly complete assignment of the chemical shifts of ^1^H, ^13^C, ^15^N and ^31^P for Gdn23.

## Methods and experiments

### RNA sample preparation

For the assignment presented herein, we used three NMR samples of Gdn23. A 660 µM natural abundance sample was purchased from Dharmacon Inc. Further two 700 µM uniformly ^13^C,^15^N-labeled samples, either in 8% D_2_O/92% H_2_O or 100% D_2_O, were prepared in house by in-vitro transcription from linearized plasmid DNA using T7 RNA polymerase (P266L mutant) (Guillerez et al. 2005). The required T7 promoter sequence, the Gdn23 sequence, the self-cleaving HDV ribozyme and the restriction sites *EcoRI* at the 5’-end and *HindIII* at the 3’-end were cloned into the pUC57 vector (GenScript) to form the plasmid. The Gdn23 sequence corresponds to the native *Escherichia coli* SugE guanidine-II riboswitch sequence to which G1 and G2 have been added at the 5’ end. This allowed efficient in-vitro transcription and should stabilize the P1 hairpin by forming the GC-terminating base pair.

The plasmid was transformed into *Escherichia coli* strain DH5α and amplified. The plasmid-DNA was purified using a large-scale DNA isolation kit (Gigaprep; Qiagen) following the manufacturer’s protocol. The linearization of plasmid was performed with *Hind* III and purified by phenol-chloroform-isoamyl alcohol extraction. In 10 mL to 15 mL preparative transcription reactions (6 h at 37 °C and 70 rpm) sufficient amounts of Gdn23 RNA (5′-ggUUUGCAGGACGACCUGCAAAC-3′) were obtained. The transcription conditions used [150 mM Tris/glutamate pH 8.1, 10% DMSO, 15 mM Mg(OAc)_2_, 20 mM dithiothreitol (DTT), 2 mM spermidine, 160 µg/mL plasmid-DNA template, 10 mM NTPs, 33.6 µg/mL homemade T7 RNA polymerase, 9.6 µg/mL homemade yeast inorganic pyrophosphatase (YIPP)] were optimized for yield and sample purity. The reaction was terminated by the addition of 150 mM EDTA, followed by RNA precipitation with ice-cold propan-2-ol. The RNA fragments were separated by 15% denaturing polyacrylamide (PAA) gel electrophoresis (5 h at 240 V) and visualized by UV shadowing at 254 nm. The Gdn23 RNA was then excised from the gel, granulated and eluted in two volumes of 0.3 M NaOAc solution through passive diffusion (overnight at room temperature and 1300 rpm). After RNA precipitation with EtOH, the residual PAA was removed by reversed-phase HPLC using a Kromasil RP 18 column and a gradient of 0–50% 0.1 M acetonitrile/triethylammonium acetate at room temperature. RNA-containing fractions were freeze-dried and subsequently dissolved in water for cation exchange by LiClO_4_ precipitation (2% in acetone). The buffer was exchanged to NMR buffer (25 mM potassium phosphate buffer, 50 mM potassium chloride, pH 6.2) multiple times using Vivaspin centrifugal concentrators (2 kDa molecular weight cut-off). The RNA folding was performed by heating the sample to 95 °C and rapidly cooling on ice. The purity of Gdn23 was verified through denaturing PAA gel electrophoresis (SI Fig. S1A, left), while the homogeneity of the folding process was monitored through native PAA gel electrophoresis (SI Fig. S1A, right), utilizing the identical RNA concentration employed in the NMR experiments.

### NMR experiments

NMR measurements were carried out at the Center for Biomolecular Magnetic Resonance (BMRZ) at the Goethe University Frankfurt using Bruker NMR spectrometers from 600 to 900 MHz, equipped with a 5 mm, z-axis gradient ^1^H/^19^F [^13^C,^15^N]-TCI prodigy probe and AV III HD console (600 MHz); a 5 mm, z-axis gradient ^1^H [^13^C,^15^N]-TCI cryo probe and AV NEO console (600 MHz), a 5 mm, z-axis gradient ^1^H [^13^C,^15^N,^31^P]-QCI cryogenic probe and AV III HD console (700 MHz); a 5 mm, z-axis gradient ^13^C [^15^N,1H]-TXO cryogenic ^13^C-optimized probe and AV III console (800 MHz); a 5 mm, z-axis gradient ^1^H [^13^C,^15^N]-TXI probe and AV NEO console (900 MHz).

All NMR experiments conducted for the resonance assignment of Gdn23 are summarized in SI Table S1 and were performed on 280 µL samples in Shigemi NMR tubes (Shigemi Inc.) at 298 K (room temperature). NMR spectra were processed and analyzed using software programs TopSpin^®^ 3.6.2 (Bruker, BioSpin, Germany) and NMRFAM-SPARKY (Lee et al. 2015). ^1^H chemical shifts were referenced to DSS as internal standard, and ^13^C, ^15^N and ^31^P chemical shifts were indirectly referenced from the ^1^H chemical shift as described earlier (Wishart et al. 1995; Maurer and Kalbitzer 1996).

### Extent of assignments and data deposition

#### Assignment strategy

The assignment strategy of Gdn23 was essentially followed in the classical way (Fürtig et al. 2003) using the NMR experiments listed in SI Table S1.

**Fig. 1:**
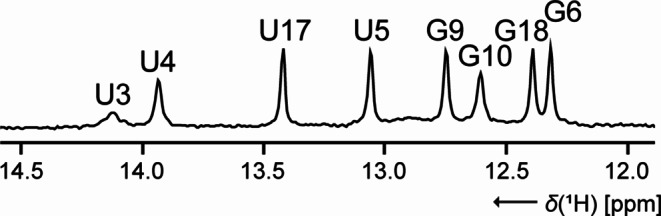
Chemical shift assignment of the base-paired imino protons for Gdn23 in 1D-^1^H spectrum detected at 20 µM RNA concentration

From the chemical shift assignment of the (stable) base-paired imino protons (89%, only G2 is missing, Fig. 1) based on 2D-^1^H,^15^N-TROSY and 2D-^1^H,^1^H-NOESY spectra, the U-C2 (100%) and -C4 (75%) as well as G-C2 and -C6 (each 43%, the guanosines of the 5’-end, G1 and G2, as well as of the loop, G13, are missing) could be assigned in the 2D-^1^H,^13^C-H(N)CO. The pyrimidine nucleobase spin pairs C5-H5 and C6-H6 were obtained completely using 2D-^1^H,^1^H-TOCSY for selective assignment of cytidine and uridine H5-H6 resonances and 2D-^1^H,^13^C-HSQC for aromatic carbon resonances. The subsequent step was to identify the chemical shifts associated with the aromatic H6/H8 and anomeric H1’ protons. As the 2D-^1^H,^1^H-NOESY could initially not be evaluated due to the aspects discussed in detail in the next section and also the 3D-^13^C-NOESY-HSQC was ambiguous, a 4D-^13^C_aromatic_,^13^C_ribose_-edited NOESY was used to ensure an unambiguous sequential assignment (SI Fig. S2). Accordingly, the assignment in 2D-^1^H,^13^C-HSQCs for H2-C2, H6-C6 and H6-C8 as well as for H1’-C1’ was completed to 100%. Further insights into the CH ribose resonance shifts (also 100%) were provided by 2D-^1^H,^13^C-CT-HSQC and 3D-(H)CCH-TOCSYs with varying mixing times. Additional ^15^N chemical shift assignments were obtained from 2D-^1^H,^15^N-HSQCs optimized for ^2^J couplings (N1 and N3 for all adenosines (expect N3 for loop nucleotide A11), N7 (100%) and N9 (92%, only G2 is missing again) for adenosines and guanosines) and the amino group region (N4-H41 and -H42 for five of six cytidines (83%), respectively. Finally, the ^31^P resonances were completely assigned using a 3D-H(C)P-CCH-TOCSY spectrum (SI Fig. S3) and verified our sequential assignment.

In conclusion, the expected assignment is largely complete with minor gaps remaining for the stem-closing nucleotides (G1, G2 and C23) and the loop nucleotides (A11-A14).

### The special challenges of the sequence

**Fig. 2:**
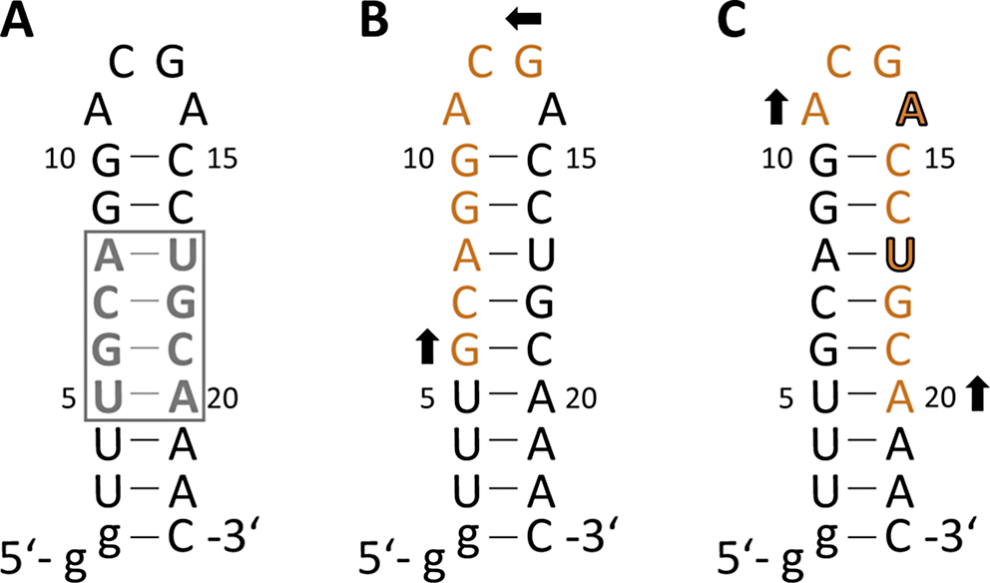
Palindromic challenges of the Gdn23 sequence from *E. Coli*. (**A**) Symmetrical base pair arrangement U5-A8 and U17-A20; (**B**) Palindromic sequence between G6 and G13; (**C**) Almost palindromic sequence between A11 and A20, except A14 and U17

An RNA sequence with 23 nucleotides is supposedly relatively easy to assign using standard 2D and, if necessary, 3D NMR experiments. A strong signal overlap is normally only to be expected for larger RNA constructs. However, this is not the case for Gdn23, as the sequence presents particular challenges. On the one hand, the stem is subject to a completely symmetrical base pair arrangement in the U5-A8 and U17-A20 region (Fig. 2A). On the other hand, there are palindromic sequence segments of nucleotides G6-G13 (Fig. 2B) and A11-A20 with the exception of A14 and U17 (Fig. 2C).

**Fig. 3:**
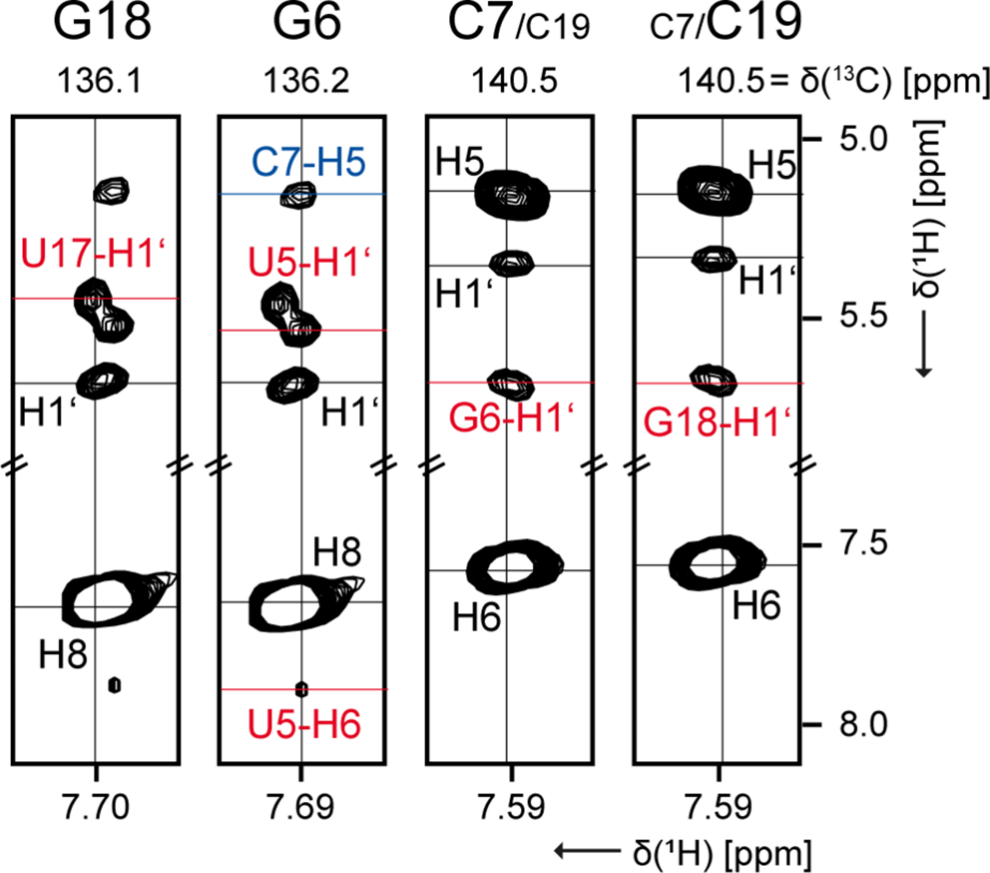
View in 3D-NOESY-HSQC for the double signals G6/G18 and C7/C19. The lettering is colored as follows: the nucleotide (n) is represented in black and the neighboring nucleotide are represented in red (n − 1) and in blue (n + 1), respectively. Experimental details are listed in SI table S1

The overlapping signals for G6/G18 and C7/C19 (Fig. 3) are particularly problematic, but they are also the key signals for the whole assignment. It is regrettable that nucleotide-specific labeling strategies are useless in this case. While it is impossible to distinguish the C8-H8 signals of G6/G18 in 2D-^1^H,^13^C-HSQC, the corresponding C1’-H1’ signals fortunately show a better resolution. Accordingly, 3D-^13^C-NOESY-HSQC reveals that the H8 resonances for G6 is high-field shifted compared to G18 (Fig. 3). This can be confirmed by the neighboring H1’ (U5 and U17), because they are affected by a different chemical environment, respectively.

**Fig. 4:**
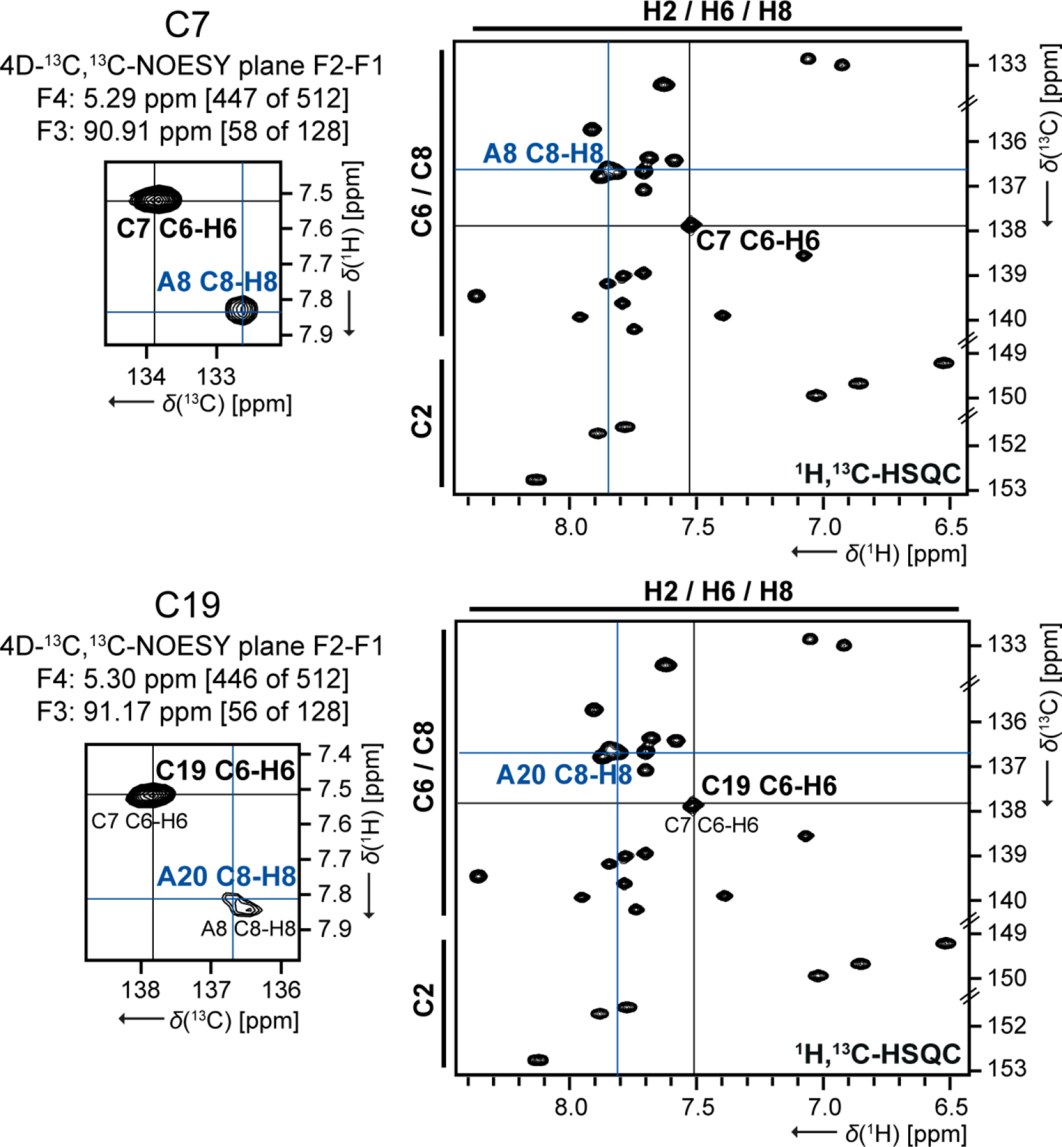
Verification of the double signal C7/C19 in 4D-^13^C_aromatic_,^13^C_ribose_-edited NOESY comparing to C1’-H1’-region of 2D-^1^H,^13^C-HSQC. The lettering is colored as follows: the nucleotide (n) is represented in black and the neighboring nucleotide are represented in red (n − 1) and in blue (n + 1), respectively. For convenience, the reference used here is not the same as the final reference. Experimental details are listed in SI table S1

In case of C7/C19, the resolution of the C1’-H1’ signals is not sufficient and the C6-H6 signals offer a little more confidence due to a general signal asymmetry. At least the 3D-^13^C-NOESY-HSQC for the H6 resonances suggests that C7 is more low-field than C19. The analysis of the F4-F3 plane of the 4D experiment does not provide the required resolution. Nevertheless, a complete sequential assignment is still possible by using the identical plane for the signals of C7 and C19 (SI Fig. S2). In contrast, analyzing the F2-F1 plane of the 4D experiment (Fig. 4) affords a crucial difference between C7 and C19, allowing the unambiguous identification of either A8 or A20 as the respective neighboring nucleotide in the 3’ direction. This confirms and supports the putative assignment based on the 3D experiment. However, the direct neighboring nucleotides of C7 and C19 have a similar chemical environment, the assignment is usually based on the overlapping C1’-H1’ signals of C7 and C19, which extends to the entire sugar assignment (C1’-H1’ to C5’-H5’/H5’’). We did not stereospecifically assign the H5’/H5’’ protons.

Taking a closer look in the 4D planes of A11 and C16 (SI Fig. S2), we note the presence of the corresponding sequential nucleotide (G10 and C15, respectively) and the non-sequential guanosine of the loop (G13). NOE-based experiments show the typical sequential walk in A-helical RNA regions while in loop regions frequently non-sequential NOEs are detected.

Another problem that must be considered with existing palindromes is that the sequence may not only be present in the monomer conformation but also in the dimer conformation. Consequently, we observed a remarkable mixture of different conformations in the 1D spectra, specifically for the imino protons if the RNA sample was not carefully refolded (SI Fig. S1B). However, the analysis of the 2D-^1^H,^13^C-HSQC and multidimensional spectra (3D, 4D) contained one set of signals enabling an effective analysis of the monomer conformation. It was only in the 2D-^1^H,^1^H-NOESY spectra that the other conformations manifested themselves as an interference factor with regard to an unambiguous assignment.

**Fig. 5:**
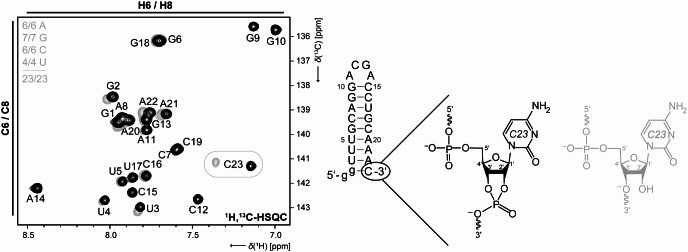
Comparison of the aromatic 2D-^1^H,^13^C-HSQC spectra for two Gdn23 samples with different 3’-ends depending on their preparation method: The transcription from a linearized plasmid with a self-cleaving HDV ribozyme forms a 2‘,3‘-cyclic phosphate (black). The transcription of the PCR product yields hydroxyl groups at the corresponding 2’- and 3’-positions of ribose (gray). The samples were also prepared in different buffers: 25 mM potassium phosphate buffer, 50 mM potassium chloride, pH 6.2 (black) and only in H_2_O without any salt (gray)

For two Gdn23 samples that differ in their preparation (second one not used for assignment reported herein), it is possible to demonstrate that the C6-H6 signal of C23 has a major deviation in the chemical shift due to the fact of different 3’-ends (Fig. 5). The phenomenon is already known in the literature (Shigematsu et al. 2018). Beyond this the helix termini are sensitive to buffer conditions because lower stem nucleotides (G1, G2-U4, and A21-C23) which also have a minor deviation in the chemical shift (Fig. 5).

In summary, we have characterized the apo-form of the binding pocket of Guanidine-II riboswitches and this serve as a starting point for NMR-based inhibitor screening.

## Electronic supplementary material

Below is the link to the electronic supplementary material.

Supplementary Material 1

## Data Availability

NMR chemical shift assignments have been submitted to BMRB with the primary access code 52714.
