# Supplementary material for: 1H, 13C, 15N and 31P chemical shift assignment of the first stem-loop Guanidine-II riboswitch from Escherichia coli
Source: Biomol NMR Assign. 2025 Feb 1;19(1):53–8. doi: 10.1007/s12104-025-10217-6 (PMC12117010; doi:10.1007/s12104-025-10217-6)
Supplement: Supplementary file 1 — Supplementary Material 1 [file 12104_2025_10217_MOESM1_ESM.docx]

**Supplementary Information**

**^1^H, ^13^C, ^15^N and ^31^P chemical shift assignment of the first stem-loop Guanidine-II riboswitch from *Escherichia Coli***


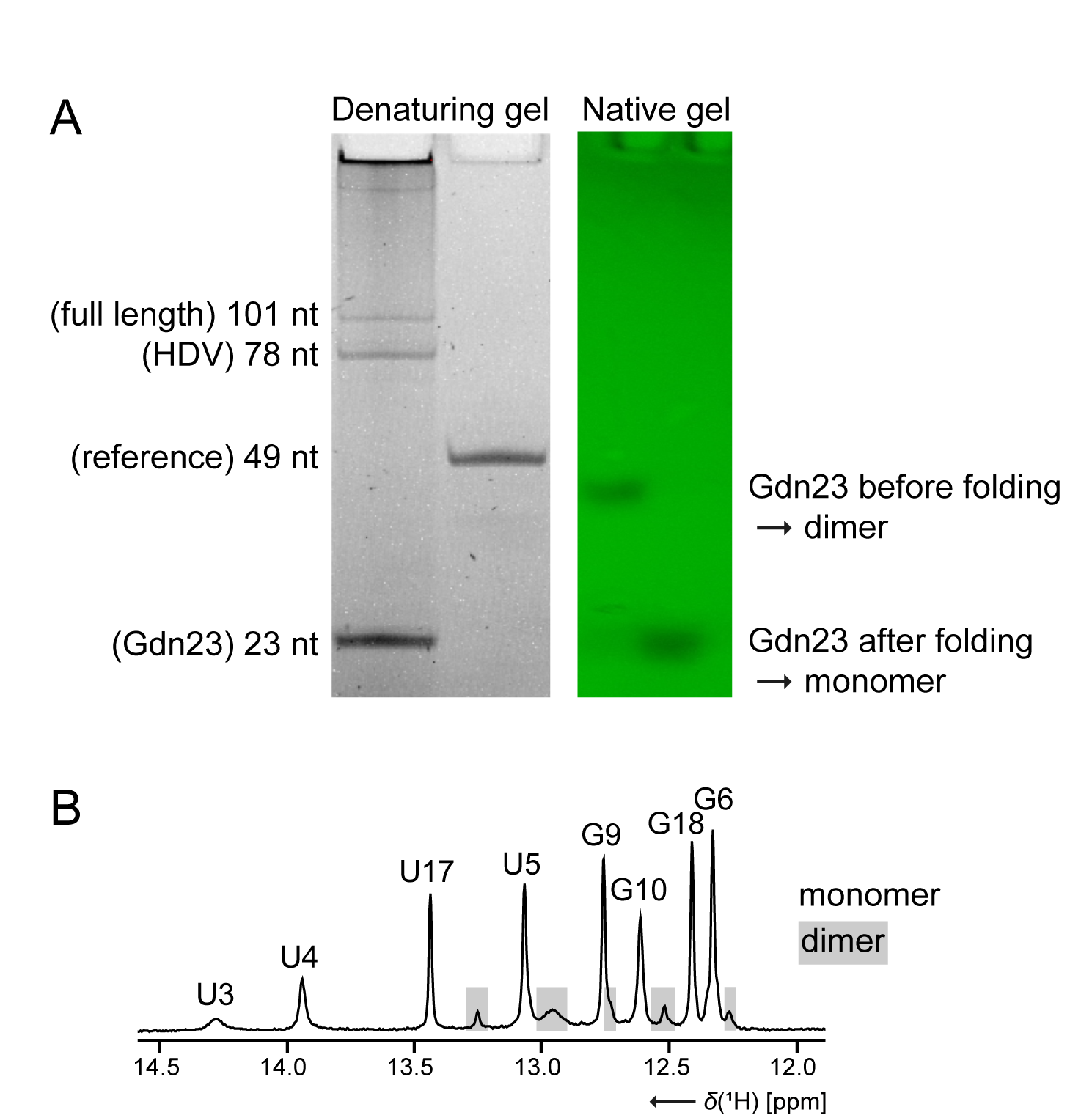


**SI Fig. S1:** Verifying Gdn23 for purity and homogeneity

A) Analytical denaturing gel electrophoresis (12%, Tris/borate/EDTA buffer, 240 V, 40 min, 10 pmol RNA) was performed prior to the purification process. Visualization by GelRed and UV light, a pure RNA band is visible at expected position (left).

The NMR sample of Gdn23 was subjected to native gel electrophoresis (15%, Tris/Acetate buffer, 20-26 mA, 35-40 V, electrical power < 1 W, water cooling, 7.5 h, 400 pmol RNA) before and after the folding process (heating to 95 °C and rapidly cooling on ice). Visualization by UV light, the gel confirms after folding a homogeneous, monomeric fold of the Gdn23 RNA (right).

B) The 1D-^1^H spectra of imino protons demonstrates the presence of distinct sets of signals which are attributed to different conformations. The high RNA concentration (700 µM) required for multidimensional NMR measurements may be a contributing factor if the RNA sample was not carefully refolded.

***SI Table S1:*** *List of NMR experiments conducted at 298 K for the resonance assignment of Gdn23.*

*Experimental parameters are given: ns = number of scans, sw = spectral width, TD = number of points in the fid, aq = acquisition time, o1-o4 = carrier frequencies on channels 1-4, rel. delay = relaxation delay. It was used a natura­­­l abundance or a uniformly ^13^C,^15^N-labeled sample. The pulse sequences were mostly optimized in-house.*

| **NMR experiment** | **Experimental parameters** |
| --- | --- |
| **2D-^1^H,^1^H-NOESY**  jump-return water suppression  natural abundance  *for iminos*  (Sklenář and Bax 1987; Sklenář 1995) | 800 MHz, ns: 256, sw(f2): 24.0 ppm, sw(f1): 15.0 ppm, TD(f2): 4096, TD(f1): 648, aq(f2): 106.5 ms, aq(f1): 26.9 ms, o1(^1^H): 4.7 ppm, rel. delay: 1.2 s, NOE mixing time: 200 ms, JR-delay: 35 µs, time: 2 d 23 h |
| **2D-^1^H,^1^H-NOESY**  watergate water suppression  natural abundance  *for aromatics*  (Piotto et al. 1992; Liu et al. 1998) | 900 MHz, ns: 160, sw(f2): 24.1 ppm, sw(f1): 15.0 ppm, TD(f2): 4096, TD(f1): 656, aq(f2): 94.2 ms, aq(f1): 24.3 ms, o1(^1^H): 4.7 ppm, rel. delay: 1.3 s, NOE mixing time: 150 ms, time: 1 d 23 h |
| **2D-^1^H,^1^H-TOCSY**  *for H5-to-H6*  (Shaka et al. 1988; Hwang and Shaka 1995) | 600 MHz, ns: 128, sw(f2): 8.8 ppm, sw(f1): 6.2 ppm, TD(f2): 1398, TD(f1): 256, aq(f2): 133 ms, aq(f1): 34 ms, o1(^1^H): 4.7 ppm, o2(^13^C): 137.0 ppm, o3(^15^N): 86.0 ppm, rel. delay: 1.0 s, TOCSY mixing time (dipsi3 spin lock): 30 ms, time: 11 h |
| **2D-^1^H,^15^N-bestTROSY**  (Solyom et al. 2013) | 800/81 MHz, ns: 16, sw(f2): 21.1 ppm, sw(f1): 24.7 ppm, TD(f2): 2018, TD(f1): 280, aq(f2): 59.7 ms, aq(f1): 70.0 ms, o1(^1^H): 4.7 ppm, o2(^13^C): 101.0 ppm, o3(^15^N): 153.0 ppm, rel. delay: 0.3 s, time: 30 min |
| **2D-^1^H,^15^N-CPMG-NOESY**  (Mueller et al. 1995; Mulder et al. 1996) | 800/81 MHz, ns: 232, sw(f2, ^1^H): 21.8 ppm, sw(f1, ^15^N): 102.8 ppm, TD(f2): 2048, TD(f1): 200, aq(f2): 58.6 ms, aq(f1): 120.0 ms, o1(^1^H): 4.7 ppm, o2(^13^C): 101.0 ppm, o3(^15^N): 116.0 ppm, rel. delay: 1.4 s, NOE mixing time: 150 ms, time: 21 h |
| **2D-^1^H,^15^N-HSQC**  *for ^2^J coupling*  (Mori et al. 1995) | 600/61 MHz, ns: 16, sw(f2, ^1^H): 10.0 ppm, sw(f1, ^15^N): 78.3 ppm, TD(f2): 1024, TD(f1): 384, aq(f2): 85.2 ms, aq(f1): 40.3 ms, o1(^1^H): 4.7 ppm, o2(^13^C): 101.0 ppm, o3(^15^N): 201.5 ppm, rel. delay: 1.0 s, coupling delay: 10 ms (^2^J_NH_: 25 Hz), time: 1 h |
| **2D-^1^H,^15^N-HSQC**  *for amino group region*  (Mori et al. 1995) | 600/61 MHz, ns: 16, sw(f2, ^1^H): 10.0 ppm, sw(f1, ^15^N): 30.8 ppm, TD(f2): 1024, TD(f1): 256, aq(f2): 85.2 ms, aq(f1): 68.3 ms, o1(^1^H): 4.7 ppm, o2(^13^C): 101.0 ppm, o3(^15^N): 86.5 ppm, rel. delay: 0.8 s, coupling delay: 2.3 ms (^2^J_NH_: 110 Hz), time: 1 h |
| **2D-^1^H,^13^C-HSQC**  A: C2/C6/C8; B: C5  (Bodenhausen and Ruben 1980) | 800/201 MHz, ns: 16, sw(f2, ^1^H): 8.3 ppm, sw(f1, ^13^C) – A: 24.0 ppm and B: 16.0 ppm, TD(f2): 1024, TD(f1) – A: 256 and B: 384, aq(f2): 76.7 ms, aq(f1) – A: 26.5 ms and B: 59.6 ms, o1(^1^H): 4.7 ppm, o2(^13^C) – A: 143.0 ppm and B: 100.0 ppm, o3(^15^N): 150.0 ppm, rel. delay: 1.0 s, INEPT transfer time –A: 1.3 ms (^1^J_CH_: 200 Hz) and B: 1.6 ms (^1^J_CH_: 155 Hz), time: 2 h |
| **2D-^1^H,^13^C-CT-HSQC**  *for C1’-C5’*  (Vuister and Bax 1992) | 600/151 MHz, ns: 4, sw(f2, ^1^H): 8.3 ppm, sw(f1, ^13^C): 38.0 ppm, TD(f2): 1024, TD(f1): 256, aq(f2): 102.4 ms, aq(f1): 22.3 ms, o1(^1^H): 4.7 ppm, o2(^13^C): 77.0 ppm, o3(^15^N): 150.0 ppm, rel. delay: 1.0 s, INEPT transfer time: 1.6 ms (^1^J_CH_: 155 Hz), constant-time period: 12.5 ms (^1^J_CC_: 80 Hz), time: 20 min |
| **2D-^1^H,^13^C-bestTROSY-H(N)CO**  *for imino-to-carbon*  (Favier and Brutscher 2011; Solyom et al. 2013) | 800/201 MHz, ns: 256, sw(f3, ^1^H): 21.1 ppm, sw(f1, ^13^C): 31.1 ppm, TD(f3): 1582, TD(f1): 256, aq(f3): 46.8 ms, aq(f1): 20.5 ms, o1(^1^H): 4.7 ppm, o2(^13^C): 157.5 ppm, o3(^15^N): 153.0 ppm, rel. delay: 0.3 s, time: 8 h |
| **3D-^13^C-NOESY-HSQC**  *for aromatics and ribose*  (Piotto et al. 1992; Sklenář et al. 1993) | 800/201/800 MHz, ns: 16, sw(f3,^1^H): 8.8 ppm, sw(f2, ^13^C): 21.0 ppm, sw(f1, ^1^H): 6.2 ppm, TD(f3): 1024, TD(f2): 88, TD(f1): 168, aq(f3): 73.1 ms, aq(f2): 10.4 ms, aq(f1): 16.8 ms, o1(^1^H): 4.7 ppm, o2(^13^C): 143.0 ppm, o3(^15^N): 154.0 ppm, rel. delay: 1 s, HC-INEPT transfer time: 1.4 ms (^1^J_CH_: 180 Hz), NOE mixing time: 300 ms, time: 3 d 22 h |
| **4D-^13^C_aromatic_,^13^C_ribose_-edited NOESY**  *for sequential assignment:* *C6/C8-to-C1’*  (Stanek et al. 2013) | 800/201/201/800 MHz, ns: 8, sw(f4, ^1^H): 9.1 ppm, sw(f3,^13^C): 16.6 ppm, sw(f2, ^13^C): 22.1 ppm, sw(f1, ^1^H): 2.8 ppm, TD(f4): 1024, TD(f3): 56, TD(f2): 64, TD(f1): 128, aq(f4): 70.2 ms; aq(f3): 8.4 ms, aq(f2): 7.2 ms, aq(f1): 28.8 ms, o1(^1^H): 4.7 ppm, o2(^13^C): 143.0 ppm, o3(^15^N): 157.0 ppm, rel. delay: 0.9 s, NOESY mixing time: 150 ms, time: 5 d 23 h |
| **3D-(H)CCH-TOCSY**  A: C1‘ to C2‘; B: C1‘ to C5‘  (Kay et al. 1993; Richter et al. 2010) | 600/151/151 MHz, ns: 8, sw(f3,^1^H): 8.8 ppm, sw(f2, ^13^C): 9.5 ppm, sw(f1, ^13^C): 35.5 ppm, TD(f3): 1024, TD(f2): 56, TD(f1): 144, aq(f3): 97.3 ms, aq(f2): 19.6 ms, aq(f1): 13.4 ms, o1(^1^H): 4.7 ppm, o2(^13^C): 76.5 ppm, o3(^15^N): 153.0 ppm, rel. delay: 1.0 s, CC-TOCSY mixing time (dipsi3 spin lock) – A: 5.4 ms and B: 16.3 ms, time – A: 21 h 50 min and B: 22 h |
| **3D-H(C)P-CCH-TOCSY**  100 % D_2_O  *for phosphorus and sequential assignment: P-to-C1’/H1’*  (Marino et al. 1995) | 700/176/283 MHz, ns: 128, sw(f3,^1^H): 5.0 ppm, sw(f2, ^13^C): 6.3 ppm, sw(f1, ^13^C): 2.7 ppm, TD(f3): 512, TD(f2): 120, TD(f1): 64, aq(f3): 73.1 ms, aq(f2): 54.0 ms, aq(f1): 41.6 ms, o1(^1^H): 4.7 ppm, o2(^13^C): 89.0 ppm, o3(^31^P): -1.3 ppm, rel. delay: 0.8 s, CC-TOCSY mixing time (dipsi3 spin lock): 18.3 ms, time: 2 d 23 h |


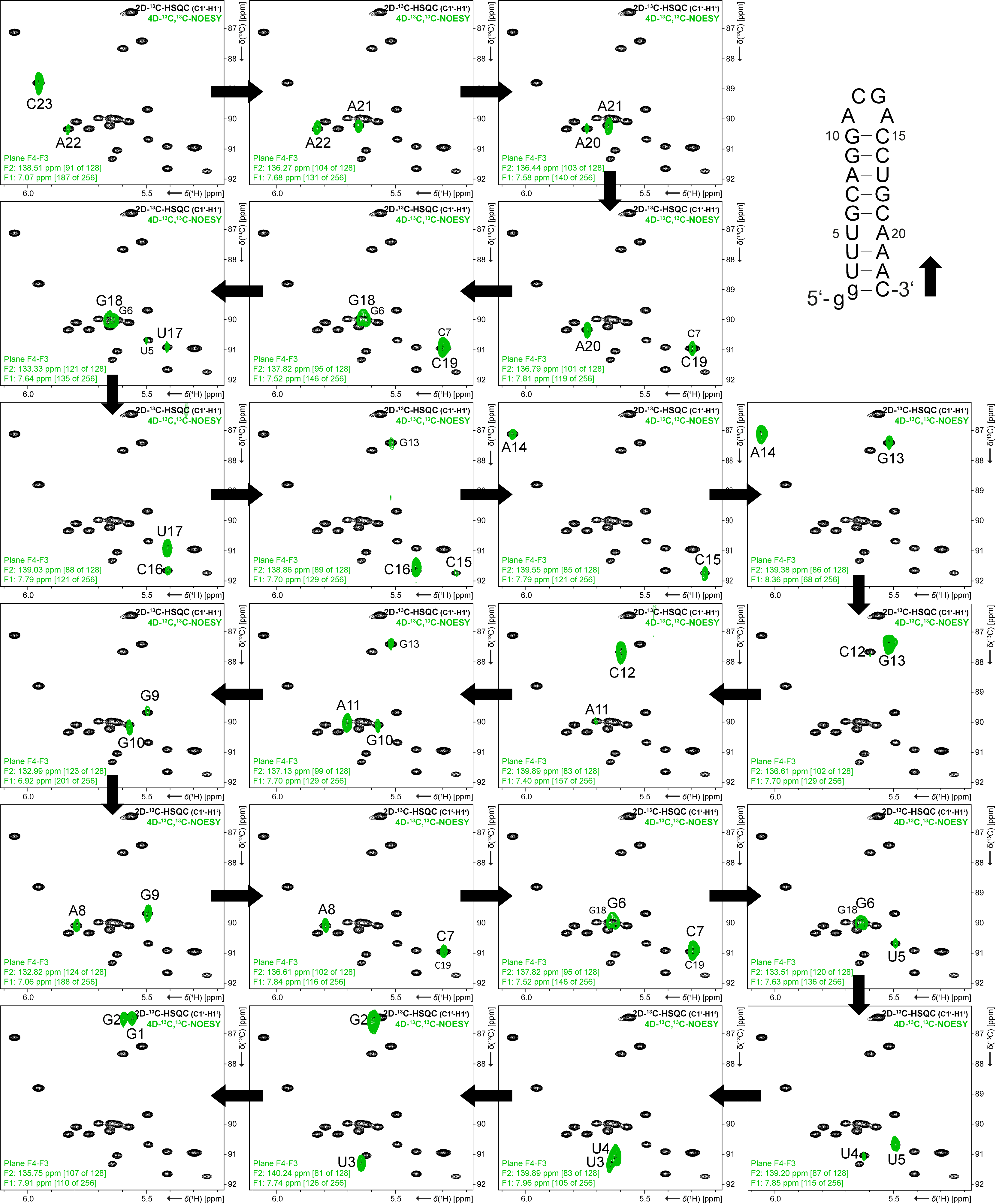


**SI Fig. S2:** Sequential assignment for Gdn23 from 4D-^13^C_aromatic_,^13^C_ribose_-edited NOESY

*The signal resonances of C6-H6/C8-H8 and C1'-H1' from the 2D-^1^H,^13^C-HSQC are correlated in the 4D, thereby enabling the sequential assignment of the adjacent nucleotides in the 3'-5' direction.*

*For this purpose, the signal resonance C6-H6/C8-H8 of a single nucleotide is used as F4-F3 plane of the 4D. Only C1'H1' signals of the own nucleotide (n) and the direct neighboring nucleotide (n-1) are visible there. The evaluation is performed by superposition the corresponding F4-F3 plane of the 4D (in green) on the C1'-H1' region of the 2D-^1^H,^13^C-HSQC (in black).*

*As a consequence of signal overlap, C7 and C19 are deployed in the same plane.*

*For convenience, the reference used here is not the same as the final reference.*

***SI Fig. S3:*** *Sequential assignment for Gdn23 from 3D-H(C)P-CCH-TOCSY*

*The phosphate backbone of Gdn23 exhibits the resonance sequence in the 5’-3’ direction with interruptions between G2 and U3 as well as A11 and C12.*


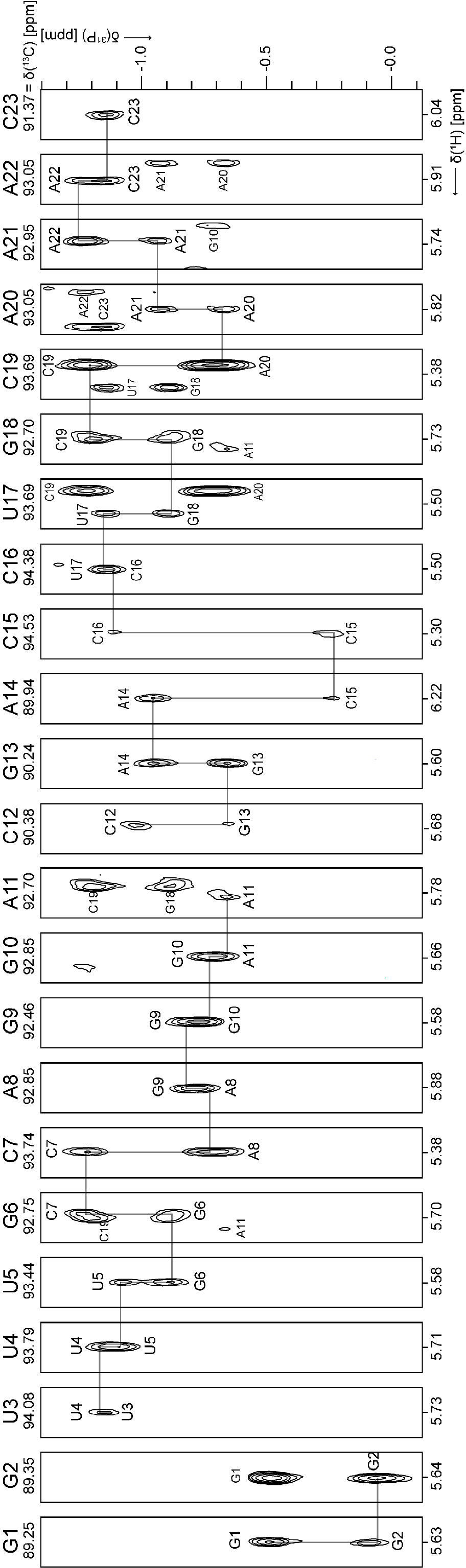


**SI References**

Favier A, Brutscher B (2011) Recovering lost magnetization: Polarization enhancement in biomolecular NMR. J Biomol NMR 49:9–15. https://doi.org/10.1007/s10858-010-9461-5

Hwang TL, Shaka AJ (1995) Water suppression that works. Excitation sculpting using arbitrary wave-forms and pulsed-field gradients. J Magn Reson Ser A 112:275–279. https://doi.org/10.1006/jmra.1995.1047

Kay LE, Xu GY, Singer AU, et al (1993) A gradient-enhanced HCCH-TOCSY experiment for recording side-chain 1H and 13C correlations in H2O samples of proteins. J Magn Reson Ser B 101:333–337. https://doi.org/10.1006/jmrb.1993.1053

Liu M, Mao X, Ye C, et al (1998) Improved WATERGATE pulse sequences for solvent suppression in NMR spectroscopy. J Magn Reson 132:125–129. https://doi.org/10.1006/jmre.1998.1405

Marino JP, Schwalbe H, Anklin C, et al (1995) Sequential correlation of anomeric ribose protons and intervening phosphorus in RNA oligonucleotides by a 1H,13C,31P triple resonance experiment: HCP-CCH-TOCSY. J Biomol NMR 5:87–92. https://doi.org/10.1007/BF00227473

Mori S, Abeygunawardana C, Johnson MO, Vanzijl PCM (1995) Improved sensitivity of HSQC spectra of exchanging protons at short interscan delays using a new fast HSQC (FHSQC) detection scheme that avoids water saturation. J Magn Reson Ser B 108:94–98. https://doi.org/10.1006/jmrb.1995.1109

Mueller L, Legault P, Pardi A (1995) Improved RNA structure determination by detection of NOE contacts to exchange-broadened amino protons. J Am Chem Soc 117:11043–11048. https://doi.org/10.1021/ja00150a001

Mulder FAA, Spronk CAEM, Slijper M, et al (1996) Improved HSQC experiments for the observation of exchange broadened signals. J Biomol NMR 8:223–228. https://doi.org/10.1007/BF00211169

Piotto M, Saudek V, Sklenář V (1992) Gradient-tailored excitation for single-quantum NMR spectroscopy of aqueous solutions. J Biomol NMR 2:661–665. https://doi.org/10.1007/BF02192855

Richter C, Kovacs H, Buck J, et al (2010) 13C-direct detected NMR experiments for the sequential J-based resonance assignment of RNA oligonucleotides. J Biomol NMR 47:259–269. https://doi.org/10.1007/s10858-010-9429-5

Shaka AJ, Lee CJ, Pines A (1988) Iterative schemes for bilinear operators; application to spin decoupling. J Magn Reson 77:274–293. https://doi.org/10.1016/0022-2364(88)90178-3

Sklenář V (1995) Suppression of radiation damping in multidimensional NMR experiments using magnetic field gradients. J Magn Reson Ser A 114:132–135. https://doi.org/10.1006/jmra.1995.1119

Sklenář V, Bax A (1987) A new water suppression technique for generating pure-phase spectra with equal excitation over a wide bandwidth. J Magn Reson 75:378–383. https://doi.org/10.1016/0022-2364(87)90046-1

Sklenář V, Piotto M, Leppik R, Saudek V (1993) Gradient-tailored water suppression for 1H-15N HSQC experiments optimized to retain full sensitivity. J Magn Reson Ser A 102:241–245. https://doi.org/10.1006/jmra.1993.1098

Solyom Z, Schwarten M, Geist L, et al (2013) BEST-TROSY experiments for time-efficient sequential resonance assignment of large disordered proteins. J Biomol NMR 55:311–321. https://doi.org/10.1007/s10858-013-9715-0

Stanek J, Podbevšek P, Koźmiński W, et al (2013) 4D Non-uniformly sampled C,C-NOESY experiment for sequential assignment of 13C,15N-labeled RNAs. J Biomol NMR 57:1–9. https://doi.org/10.1007/s10858-013-9771-5

Vuister GW, Bax A (1992) Resolution enhancement and spectral editing of uniformly 13C-enriched proteins by homonuclear broadband 13C decoupling. J Magn Reson 98:428–435. https://doi.org/10.1016/0022-2364(92)90144-V
